# Coercion and Its Discontents: The Promise and Peril of Increasingly Restrictive of Vaccination Mandates: Comment on "Convergence on Coercion: Functional and Political Pressures as Drivers of Global Childhood Vaccine Mandates"

**DOI:** 10.34172/ijhpm.2023.7617

**Published:** 2023-06-13

**Authors:** James Colgrove

**Affiliations:** Department of Sociomedical Sciences, Mailman School of Public Health, Columbia University, New York City, NY, USA

**Keywords:** Immunization, Public Health, Ethics

## Abstract

Attwell and Hannah present a cogent analysis of why policy-makers in four jurisdictions chose to use coercive approaches to increase vaccination rates between 2015 and 2017. Their study calls attention to the challenging political calculations that are necessary when choosing between coercion and persuasion to increase vaccine uptake. Further research is needed on the consequences of making a mandatory vaccination policy more restrictive, in order to better understand the backlash and resistance such a strategy may provoke. Although one reason that policy-makers may choose a coercive approach is that it is cheaper and easier to implement than a persuasive one, sociopolitical trends and backlash related to the COVID-19 pandemic may make coercive policies more politically risky in the coming years.

 To what extent should individual liberty be constrained in order to prevent the spread of illness and safeguard the common welfare? This question, a central one in public health, is especially salient in the realm of policies that aim to increase vaccine uptake by imposing sanctions or penalties on people who decline immunization for themselves or their children. Such policies can be made more or less coercive through the number of vaccines they require, the types of sanctions they impose, and the exemption provisions they make for people who do not wish to be vaccinated.^1^ Governments may also forego coercion entirely and rely instead on persuasive and educational approaches to foster vaccine acceptance.

 Policy-makers around the world have adopted divergent strategies for maximizing vaccine uptake in their populations, and much remains to be understood about when and why they choose one approach over another. The analysis of Attwell and Hannah is therefore timely and important.^2^ The authors examine the surprising and somewhat counterintuitive policy convergence in four jurisdictions (Australia, California, France, and Italy) that made compulsory vaccination more restrictive between 2015 and 2017, either by increasing the number of required vaccines or narrowing the circumstances under which people may opt out. Attwell and Hannah’s analysis usefully distinguishes between functional and political dimensions of policy issues. In explaining why governments moved in a more coercive direction, they identify perceptions among policy-makers that the immunization system was not achieving its desired goal of high uptake (a functional problem) as well as public perceptions that vaccine-refusing parents were irresponsible and dangerous to others (a political problem). The authors’ rigorous qualitative analysis of key informant interviews, using the comparative case study method, allows for an especially detailed and nuanced understanding of these issues.

 The findings of Attwell and Hannah speak to the challenging calculations that policy-makers must undertake when considering a persuasive or coercive approach, especially regarding costs and benefits that may be difficult to calculate or incommensurate with each other.

## Weighing the Pros and Cons of Coercion and Persuasion

 Coercive policies may be defended on both pragmatic and ethical grounds. A large empirical literature supports the effectiveness of compulsory approaches in increasing vaccine use and reducing incidence of contagious diseases. Simply put, mandates work. Furthermore, they are efficient: unlike persuasive and educational media campaigns, which are difficult to craft and may produce only slow or incremental results, mandates can achieve rapid increases in vaccine uptake and control of contagious diseases, thereby freeing up resources that can be used to advance other public health goals. Attwell and Hannah’s analysis succinctly presents the pragmatic justification for choosing mandates: “Buttressing a non-coercive vaccination regime requires multiple policy instruments to cultivate social trust,a project requiring a much longer timescale and continual inputs.”

 Several ethical principles support the use of coercive measure to achieve high vaccine uptake. These include individual obligations of non-maleficence; the government’s right to prevent members of society from harming each other (Mill’s harm principle); the government’s duty to protect vulnerable people in the population; and the goal of preventing a free rider problem in which non-vaccinators enjoy the benefits of herd immunity, resulting in the benefits and burdens of vaccination not being equitably distributed.^3^

 Objections to coercion may likewise be made on both ethical and pragmatic bases. Critics argue that coercion is ethically impermissible because it violates individual and parental autonomy. The principle of least restriction dictates that in choosing among effective policy approaches, those that are least burdensome to individual liberty are always preferable *a priori*, and that mandates should be used only when there is evidence that voluntary means would be insufficient to achieve the goal (a point on which many advocates of compulsory vaccination also agree).

 A further criticism, empirical rather than normative, is that coercion is counterproductive and undermines vaccine acceptance by inflaming resistance, antagonizing people who might otherwise be persuaded to accept vaccines, and eroding trust in public health authority and government authority more generally.^4^ Because of concerns about backlash, even some proponents of mandates advocate for “softer” forms of coercion that allow ample opportunities for people opposed to vaccination to opt out.^5^

 Measuring the backlash (if any) caused by coercive vaccination policies, and precisely identifying the nature and magnitude of unintended consequences, is critically important. Under what circumstances are mandates more or less likely to be met with opposition? What is the nature of these objections, and what forms does resistance take? Real-time, prospective studies are needed to evaluate the consequences of policy changes. Researchers can take advantage of “natural experiments” within and across jurisdictions where changes do or do not occur. Such studies have been useful, for example, in tracking the consequences of changes to immunization laws in California^6^ and Washington State.^7^ Since vaccine hesitancy and resistance are highly context-dependent, research should seek to understand the complex interplay of factors affecting the acceptability of a vaccine mandate, such as the severity and transmissibility of the disease and the actual and perceived safety and efficacy of the vaccine.

 In addition to intended changes resulting directly from a coercive policy, research should seek to measure a broad range of indirect outcomes and knock-on effects, including changes in public attitudes, beliefs, and trust in government and the public health system, as well as any potential negative effects such as lawsuits, opposing legislation, protests, and adverse media coverage.

## The Past, Present, and Future of Coercion

 The historical record provides many examples of backlash resulting from coercive approaches. In Europe and the Americas in the 19th and early 20th centuries, the legal enforcement of smallpox vaccination triggered vociferous, sometimes violent opposition. Indeed, the origins of the modern-day anti-vaccination movement can be traced to the British government’s enactment of increasingly coercive public health laws that required the vaccination of infants against smallpox and levied fines against parents who refused.^8^ In other countries, including the United States, Canada, Brazil, and Germany, mandatory vaccination led to protests, riots, lawsuits, and legislative battles.^9^ These events suggest that mass opposition is more likely under conditions of pre-existing political mistrust, and more successful when opponents are able to form alliances with other civil society organizations.

 The findings of Attwell and Hannah demonstrate there was momentum in the direction of strong vaccination mandates during the 2010s. These findings are consistent with other evidence from US states. Although there was an ongoing tug-of-war in the early 2000s in numerous US state legislatures over whether vaccination policy should be more or less coercive, opponents of mandatory vaccination made little headway.^10^ In the wake of the 2015 measles outbreak, both the American Medical Association and the American Academy of Pediatrics called upon states to eliminate non-medical exemptions from vaccination laws, and several U.S. states followed California’s lead in curtailing exemptions.

 However, since the time period studied by Attwell and Hannah (2015-2017), we have seen the acceleration of sociopolitical trends that may prove to be inimical to compulsory vaccination policies. The “post-truth era” that has been fostered and exacerbated by social media, and the resulting “infodemic,” creates an environment in which the safety and efficacy of vaccines are increasingly called into question. The global rise in right-wing populism and autocracy, characterized by attacks on civil society institutions, government social welfare programs, and scientific elites, poses a threat to public health in general and the use of compulsory vaccination laws in particular.^11^

 Perhaps most consequentially, the COVID-19 pandemic may have damaged the standing and credibility of public health institutions. COVID-19 gave anti-vaccination activists globally a new arena in which to advance their views and spread misinformation. The prospect of mandatory COVID-19 vaccination sparked mass protests in numerous countries.^12^ The pandemic also unleashed a fierce backlash against public health authority in general and the concept of mandatory vaccination in particular. In the United States, governmental efforts to control the spread of COVID-19 through restrictive measures such as mask mandates, quarantines, and business closings inflamed anti-government attitudes and led to threats, verbal abuse, and sometimes violent attacks on public health officials.^13^

 What is most concerning about protests against COVID-19 vaccine mandates is the possibility that they may have spillover effects that undermine other, well-established immunization laws. In the wake of political backlash over COVID-19 vaccine mandates, legislators in several US states have introduced bills to weaken or eliminate school vaccination requirements that have protected communities for decades from a host of once-common diseases.^14^

 Attwell and Hannah argue that introducing or strengthening a coercive approach may be an attractive option for policy-makers because mandates are “relatively cheap and easy compared to other forms of intervention for increasing vaccination rates.” There is reason to believe that in the coming years, mandates may become costlier—not in economic terms, but in political terms, with costs in the form of time-consuming and divisive legislative and legal battles. Such risks are not a reason to forego coercion, but they do indicate that policy-makers must proceed with caution and carefully weigh both benefits and burdens.^15^

 Our continued protection from infectious diseases depends on our ability to craft immunization policy that is effective, ethical, and politically acceptable. The questions raised by compulsory vaccination around liberty and community protection are central to democratic societies. Striking the right balance between coercion and persuasion is critical not just for public health, but for the health of our civic democracy.

## Ethical issues

 Not applicable.

## Competing interests

 Author declares that he has no competing interests.
